# Association of daily lung condition in COPD patients with wearable speech and physiological data

**DOI:** 10.1038/s41598-025-14171-0

**Published:** 2025-09-29

**Authors:** Sejal Bhalla, Deshang Kong, Salaar Liaqat, Daniyal Liaqat, Robert Wu, Andrea Gershon, Eyal de Lara, Alex Mariakakis

**Affiliations:** 1 Computer Science, University of Toronto, Toronto, ON Canada; 2 University Health Network, Toronto, ON Canada; 3 Sunnybrook Health Sciences Centre, Toronto, ON Canada

**Keywords:** COPD, Physiological data, Continuous health sensing, Statistics, Information technology, Respiration

## Abstract

Chronic obstructive pulmonary disease (COPD) is a prevalent condition that imposes significant burden on patients and healthcare systems, with COPD exacerbations being a leading cause of hospitalizations and mortality worldwide. Continuous monitoring of lung function is crucial for effective management, yet traditional methods are often cumbersome and require clinic visits. Wearable technology offers a non-invasive alternative, enabling the monitoring of physiological parameters in real-world settings. In this study, we analyzed free-living speech data collected via smartwatches from 18 COPD patients over an average period of 198.9 ± 122.6 days. Utilizing linear mixed-effects models, we discovered that phonation features are negatively associated with adverse COPD outcomes, while prosodic speech features show a positive correlation with increased exacerbation risk. Further analysis revealed that these associations are significantly moderated by physiological covariates such as heart rate variability and physical activity levels. These findings highlight the complex interplay between respiratory function, autonomic regulation, and vocal production, suggesting that the integration of speech analysis with physiological monitoring through wearables can lead to the development of composite digital biomarkers of impaired lung function.

## Introduction

Chronic obstructive pulmonary disease (COPD) is the third-leading cause of death worldwide, causing 3.3 million deaths in 2019^1^. It is characterized by progressive airflow obstruction that results in respiratory symptoms like increased coughing and sputum production. COPD has both short- and long-term impacts on health. In the short term, symptoms can flare to the point of exacerbations, during which patients can experience decreased physical reserve and impaired quality of life^2–4^. In the long term, the underlying lung inflammation and subsequent exacerbations lead to multiple medical and psychological comorbidities that increase disease burden and the likelihood of hospitalization, ultimately contributing to increased mortality and an ever-growing healthcare cost^5,6^.

The chronic nature of COPD necessitates regular supervision to enable timely interventions that could reduce exacerbations, highlighting the need for convenient methods of continuous symptom monitoring. Although spirometry and symptom diaries have been studied to this end^7–10^, they only provide brief episodic glimpses into patients’ health. COPD symptoms can worsen at any moment, so remote monitoring of physiological and behavioral data extracted from wearables has emerged as a promising approach to comprehensively observe patients’ experiences in free-living conditions.

Beyond data related to physical activity and heart rate^11–14^, there has been growing interest in acoustic biomarkers of COPD because of their direct correlation with airflow limitation^15–18^. Speech production is a complex motor task that involves the precise coordination of orofacial, laryngeal, pharyngeal, and respiratory muscles. As a person speaks, airflow from the lungs gets modulated by the vocal folds in the larynx and is further shaped by the articulators within the vocal tract to produce sound. Given the critical role of respiratory support in this process, any impairment, such as that seen in COPD patients, can lead to notable alterations in vocal characteristics.

Prior studies have examined the feasibility of analyzing speech recordings to infer COPD outcomes such as respiratory symptoms^16^ and lung function metrics^19,20^. These efforts have often involved prompted speech such as sustained vowels^21,22^ or scripted passages of text^19,22,23^. Since individuals may modulate their voice when instructed to perform specific tasks, prompted speech may not capture the full range of articulatory and respiratory behaviors relevant to disease severity. These studies have also been performed in stable indoor environments, which minimizes confounding variables at the cost of ignoring the complex acoustic and physiological conditions encountered during daily life. Passive continuous monitoring throughout the day entails ambient noise, conversational variability, and varying speaking contexts, all of which may obscure subtle vocal changes. To this end, Sedaghat et al.^24^ proposed an end-to-end machine learning approach for inferring daily symptom severity from passively collected speech, and our recent work on the PulmoListener system^25^ improved upon those results using deep learning. However, both of these works overlooked the influence of other factors that could have been related to symptom worsening and voice modulation, namely physical activity and stress.

The study presented in this paper addresses this shortcoming. After extracting interpretable vocal parameters from free-living speech captured using off-the-shelf smartwatches, we examine how those parameters correlate with daily self-reported symptoms and occurrences of exacerbations. Furthermore, we extend our investigation to include additional variables that may be associated with exacerbations, including step count and heart rate variability. To our knowledge, this is the first study to statistically analyze the relationship between natural speech patterns, physical activity, and heart rate variability in predicting COPD outcomes. By examining these factors concurrently, we aim to provide comprehensive insights into the multifaceted nature of COPD and contribute to the development of more effective monitoring and management strategies.

**Table 1:** Participant characteristics and vital signs of COPD patients (*IQR* interquartile range, *SD* standard deviation).

**Participant characteristics (N = 18)**	
Male, n (%)	12 (66.67%)
Age in years, mean $\pm$ SD	67.3 $\pm$ 8.8
Home oxygen use, n (%)	7 (38.9%)
Hospital admissions in last 12 months, median (IQR)	2 (0–10)
Days enrolled in the study, median (IQR)	187.5 (24–408)
Comorbidities, n (%)	
Connective tissue disease	2 (11.1%)
Congestive heart failure	2 (11.1%)
Diabetes	1 (5.6%)
Mild liver disease	1 (5.6%)
Peripheral vascular disease	1 (5.6%)
Ulcer disease	1 (5.6%)
Smoking history, n (%)	
Current	4 (22.2%)
Never	2 (11.1%)
Ex-smoker	12 (66.7%)
COPD outcomes	
Daily COPD symptom score^26,27^, mean $\pm$ SD	1.3 $\pm$ 2.9
Experienced at least one exacerbation, n (%)	6 (33.3%)
Exacerbation days, mean $\pm$ SD	12.8 $\pm$ 36.3
Wearable data	
Days with speech, mean $\pm$ SD	45.1 $\pm$ 50.8
Daily speech duration in minutes, mean $\pm$ SD	122.6 $\pm$ 97.4
Days with HR data, mean $\pm$ SD	85.5 $\pm$ 66.9
HR in beats per minute, mean $\pm$ SD	86.6 $\pm$ 15.9
Days with step count data, mean $\pm$ SD	136.7 $\pm$ 86.4
Daily step count, mean $\pm$ SD	1609 $\pm$ 2069

## Results

### Participant characteristics

The characteristics of our patient cohort are summarized in Table 1. The analysis was performed with 18 patients diagnosed with COPD, 12 (66.7%) of whom were male. The participants were between 55 and 93 years old (average: 67.3 $\pm$ 8.8). The majority had a smoking history, with 12 (66.7%) being ex-smokers and 4 (22.2%) being active smokers. Comorbidities were also prevalent among the participants, with 44.5% reporting at least one comorbidity. The most commonly reported COPD symptoms during the study were worsening breathlessness (15 patients, 83.3%), worsening cough (13 patients, 72.2%), and cold (12 patients, 66.7%). According to the London COPD Cohort Daily Symptom Questionnaire^26,27^, 6 patients (33.3%) experienced an exacerbation at least once during the study by recording a score exceeding 6 on two or more consecutive days.

### Univariate associations between speech and COPD outcomes

**Table 2:** The results of the univariate linear mixed-effects models that were generated to estimate relationships between COPD outcomes and free-living speech characteristics.

Category	Feature	Symptom score $\beta$-coefficient [95% CI]	Exacerbation Odds ratio [95% CI]
Phonation features	HNR	− 0.03 [− 0.24, 0.17]	1.35 [0.93, 1.98]
	jitter	− 0.20 [− 0.44, 0.03]	0.44 [0.25, 0.77]**
	absolute jitter	− 0.18 [− 0.34, − 0.01]*	0.59 [0.42, 0.83]**
	ddp jitter	− 0.19 [− 0.41, 0.03]	0.44 [0.23, 0.83]*
	ppq5 jitter	− 0.16 [− 0.37, 0.05]	0.43 [0.24, 0.78]**
	rap jitter	− 0.19 [− 0.41, 0.03]	0.44 [0.23, 0.83]*
	shimmer	− 0.21 [− 0.40, − 0.01]*	0.54 [0.37, 0.78]**
	apq3 shimmer	− 0.19 [− 0.38, − 0.00]*	0.56 [0.39, 0.81]**
	apq5 shimmer	− 0.09 [− 0.28, 0.10]	0.63 [0.46, 0.87]**
	apq11 shimmer	− 0.07 [− 0.26, 0.12]	0.59 [0.41, 0.87]**
	dda shimmer	− 0.19 [− 0.38, − 0.00]*	0.56 [0.39, 0.81]**
Prosodic features	mean F0	0.10 [− 0.11, 0.30]	1.72 [1.15, 2.57]**
	std F0	0.11 [− 0.10, 0.32]	1.62 [1.04, 2.53]*
	mean F1	− 0.05 [− 0.31, 0.20]	1.21 [0.67, 2.18]
	mean F2	− 0.24 [− 0.48, − 0.01]*	0.61 [0.35, 1.06]
	mean F3	− 0.22 [− 0.46, 0.02]	0.77 [0.50, 1.18]
	mean F4	− 0.15 [− 0.36, 0.06]	0.77 [0.50, 1.18]
	mean formant	0.04 [− 0.43, 0.51]	0.76 [0.45, 1.29]
	delta_f	− 0.24 [− 0.49, 0.02]	0.75 [0.47, 1.21]
	fdisp	− 0.19 [− 0.41, 0.03]	0.66 [0.40, 1.09]
	fitch_vtl	0.04 [− 0.22, 0.30]	0.82 [0.47, 1.41]
	mff	− 0.12 [− 0.39, 0.15]	0.94 [0.56, 1.61]
	pF	− 0.20 [− 0.67, 0.26]	0.98 [0.66, 1.46]
	vtl_delta_f	0.23 [− 0.02, 0.48]	1.32 [0.84, 2.07]
Syllabic nuclei features	pause rate	− 0.07 [− 0.24, 0.11]	1.02 [0.67, 1.54]
	phonation time	0.08 [− 0.09, 0.26]	0.90 [0.65, 1.24]
	speech rate	0.02 [− 0.23, 0.27]	0.93 [0.61, 1.42]

Significant model results are indicated as follows: $\text{* }p <.05$, $\text{** }p <.01$, $\text{*** }p <.001$. Refer to Table 6 for definitions of feature names and acronyms.

**Table 3:** The results of the multivariate linear mixed-effects models that were generated to estimate (a) daily symptom score and (b) likelihood of exacerbation. The models included significant speech features and their interaction effects with heart rate variability metrics and daily step count. Significant model results are indicated as follows: $\text{* }p < .05$, $\text{** }p < .01$, $\text{*** }p < .001$. Refer to Table 6 for definitions of feature names and acronyms.

Explanatory variables	Symptom score $\beta$-coefficient [95% CI]
(a) Symptom Score	
PA(steps)	− 0.05 [− 0.28, 0.18]
HRV(mean NN)	0.10 [− 0.16, 0.36]
HRV(mean NN):PA(steps)	− 0.13 [− 0.39, 0.13]
HRV(SDNN)	− 0.07 [− 0.29, 0.15]
HRV(pNN20)	0.38 [0.06, 0.71]*
HRV(pNN20):PA(steps)	0.30 [− 0.09, 0.70]
absolute jitter	0.15 [− 0.14, 0.45]
absolute jitter:PA(steps)	− 0.14 [− 0.50, 0.21]
absolute jitter:HRV(mean NN)	0.22 [− 0.15, 0.58]
absolute jitter:HRV(mean NN):PA(steps)	0.14 [− 0.36, 0.63]
absolute jitter:HRV(SDNN)	− 0.06 [− 0.39, 0.27]
absolute jitter:HRV(SDNN):PA(steps)	− 0.07 [− 0.48, 0.34]
absolute jitter:HRV(pNN20)	− 0.50 [− 0.99, − 0.01]*
absolute jitter:HRV(pNN20):PA(steps)	− 0.27 [− 1.00, 0.46]
apq3 shimmer	− 0.22 [− 0.53, 0.09]
apq3 shimmer:PA(steps)	0.20 [− 0.21, 0.61]
apq3 shimmer:HRV(mean NN)	− 0.25 [− 0.63, 0.13]
apq3 shimmer:HRV(mean NN):PA(steps)	− 0.21 [− 0.77, 0.34]
apq3 shimmer:HRV(SDNN)	− 0.06 [− 0.45, 0.32]
apq3 shimmer:HRV(SDNN):PA(steps)	− 0.15 [− 0.70, 0.40]
apq3 shimmer:HRV(pNN20)	0.53 [0.03, 1.02]*
apq3 shimmer:HRV(pNN20):PA(steps)	0.46 [− 0.32, 1.24]
mean F2	− 0.28 [− 0.52, − 0.04]*
mean F2:PA(steps)	0.12 [− 0.24, 0.47]
mean F2:HRV(mean NN)	− 0.31 [− 0.61, − 0.01]*
mean F2:HRV(mean NN):PA(steps)	− 0.24 [− 0.66, 0.19]
mean F2:HRV(SDNN)	− 0.06 [− 0.33, 0.20]
mean F2:HRV(SDNN):PA(steps)	− 0.30 [− 0.69, 0.08]
mean F2:HRV(pNN20)	0.03 [− 0.45, 0.51]
mean F2:HRV(pNN20):PA(steps)	0.39 [− 0.36, 1.13]

Explanatory variables	Exacerbations Odds ratio [95% CI]
(b) Exacerbations	
PA(steps)	1.03 [0.40, 2.68]
HRV(mean NN)	0.36 [0.13, 1.00]*
HRV(SDNN)	0.70 [0.31, 1.56]
HRV(pNN20)	1.04 [0.32, 3.36]
HRV(SDNN):PA(steps)	0.88 [0.33, 2.36]
HRV(pNN20):PA(steps)	1.43 [0.34, 5.99]
HRV(meanNN):PA(steps)	0.97 [0.37, 2.53]
absolute jitter	2.65 [0.75, 9.42]
absolute jitter:HRV(pNN20)	0.86 [0.37, 1.98]
absolute jitter:HRV(mean NN):PA(steps)	3.82 [1.10, 13.27]*
apq3 shimmer	1.39 [0.45, 4.27]
apq3 shimmer:PA(steps)	2.80 [0.51, 15.27]
apq3 shimmer:HRV(mean NN)	3.73 [0.99, 14.01]
apq3 shimmer:HRV(SDNN)	1.01 [0.25, 4.08]
apq3 shimmer:HRV(SDNN):PA(steps)	0.16 [0.03, 0.87]*
apq11 shimmer	0.76 [0.29, 1.98]
apq11 shimmer:PA(steps)	2.74 [0.57, 13.06]
apq11 shimmer:HRV(mean NN)	0.48 [0.18, 1.31]
apq11 shimmer:HRV(mean NN):PA(steps)	0.49 [0.11, 2.10]
apq11 shimmer:HRV(SDNN)	1.36 [0.48, 3.84]
apq11 shimmer:HRV(pNN20):PA(steps)	1.02 [0.31, 3.41]
ddp jitter	0.11 [0.02, 0.69]*
ddp jitter:HRV(SDNN)	0.53 [0.10, 2.78]
ddp jitter:HRV(mean NN)	0.08 [0.02, 0.38]**
ddp jitter:PA(steps)	0.13 [0.01, 1.69]
mean F0	1.81 [0.56, 5.90]
mean F0:HRV(mean NN)	0.23 [0.06, 0.93]*
mean F0:HRV(SDNN)	0.92 [0.21, 3.93]
mean F0:HRV(SDNN):PA(steps)	1.24 [0.26, 5.90]
mean F0:HRV(pNN20)	1.10 [0.22, 5.57]
mean F0:HRV(pNN20):PA(steps)	1.23 [0.23, 6.58]
mean F0:PA(steps)	1.08 [0.18, 6.32]
std F0:HRV(mean NN)	2.21 [0.54, 9.14]
std F0:HRV(mean NN):PA(steps)	1.49 [0.38, 5.91]
std F0:HRV(SDNN)	0.47 [0.11, 2.02]
std F0:HRV(pNN20)	1.40 [0.35, 5.61]
std F0:PA(steps)	0.47 [0.05, 4.60]

Table 2 shows the results of the linear mixed−effects models between speech features and either daily symptom score or exacerbations. There were no significant associations between syllabic nuclei features and either COPD outcome. Among phonation features, higher values of jitter and shimmer were associated with a decrease in daily symptom score (absolute jitter: β = − 0.18, $p < .05$, 95% CI [− 0.34, − 0.01]; apq3 shimmer: β = − 0.21, $p < .05$, 95% CI [− 0.40, − 0.01]) and lower odds of exacerbation (jitter: OR $= 0.44$, $p < .01$, 95% CI [0.25, 0.77]; shimmer: OR $= 0.54$, $p < .01$, 95% CI [0.37, 0.78]). Among prosodic features, the mean value of the second formant showed a statistically significant negative association with daily symptom score (mean F2: β = − 0.24, $p < .05$, 95% CI [− 0.48, − 0.01]). Conversely, higher mean and standard deviation values of the fundamental frequency were associated with higher odds of exacerbation (mean F0: OR $= 1.72$, $p < .01$, 95% CI [1.12, 2.57]; std F0: OR $= 1.62$, $p < .05$, 95% CI [1.04, 2.53]).

### Multivariate associations between speech and COPD outcomes

Table 3a shows the results of the multivariate linear mixed−effects model between speech features and physiological covariates with respect to daily symptom score. This analysis only includes features that demonstrated significant univariate relationships without multicollinearity in relation to other features. The association with prosodic features was robust with respect to the physiological covariates. Similar to the corresponding univariate model, higher mean values of the second formant were associated with statistically significant decreases in daily symptom scores (mean F2: β = − 0.24, $p < .05$, 95% CI [− 0.48, − 0.01]). The same could not be said for the phonation features, jitter and shimmer, since their relationship with the symptom score lost its significance. The model also revealed significant interactions between heart rate variability (HRV) metrics and phonation features, including HRV(pNN20) with jitter, HRV(pNN20) with shimmer, and HRV(mean NN) with mean F2.

**Table 4:**
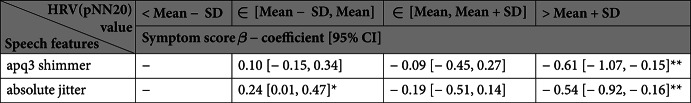
The results of the univariate linear mixed−effects models generated to estimate daily symptom score and stratified by different levels of HRV(pNN20) for speech features.

Note that the column for HRV(pNN20) < Mean - SD is empty due to a lack of data in that quadrant. Significant model results are indicated as follows: $\text{* }p <.05$, $\text{** }p <.01$, $\text{*** }p <.001$.

**Fig. 1:**
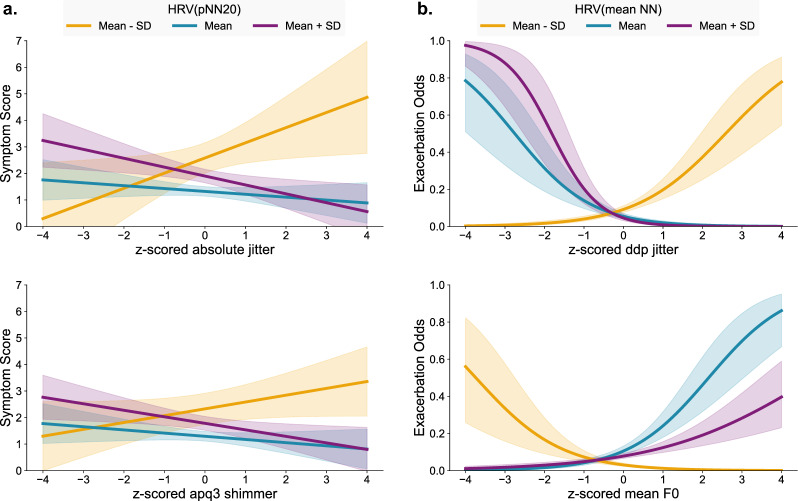
Interactions between HRV and z-score normalized speech features for (**a**) symptom score and (**b**) likelihood of exacerbation. The shaded regions surrounding each curve denote the 95% confidence intervals.

Since HRV(pNN20) emerged as a common moderator among the significant two-way interaction terms involving absolute jitter and apq3 shimmer in the symptom score model, we applied a moderation analysis to assess how varying levels of autonomic function according to HRV(pNN20) affected the direction and strength of association between speech features and COPD outcomes. The results of this analysis are shown in Table 4 and visualized in Fig. 1a. Both jitter and shimmer had a negative association with symptom score in the presence of high HRV(pNN20). However, an increase in jitter was associated with a statistically significant increase in symptom score when lower HRV(pNN20) values were measured.

**Table 5:**
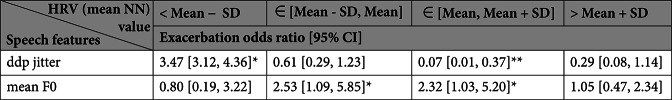
The results of the univariate linear mixed-effects models generated to estimate the likelihood of exacerbation and stratified by different levels of HRV(mean NN) for speech features.

Significant model results are indicated as follows: $\text{* }p <.05$, $\text{** }p <.01$, $\text{*** }p <.001$.

Table 3b shows the results of the multivariate linear mixed-effects model between speech features and physiological covariates with respect to exacerbations. None of the speech features retained the statistical significance of their association. However, multiple two-way interactions across different categories of speech features were found to be significant: HRV(SDNN) with std F0, HRV(mean NN) with shimmer, HRV(pNN20) with shimmer, PA(steps) with jitter, and PA(steps) with mean F0. Table 5 illustrates the relationship between speech features and exacerbations when stratified by different levels of HRV(mean NN), as that was the most prominent HRV metric for this model. These significant interaction effects are visualized in Fig. 1b. This analysis allowed us to assess whether the strength and direction of these associations vary across levels of autonomic function, as measured by HRV(mean NN). The results show that lower values of jitter are associated with lower odds of exacerbation during periods with low HRV(mean NN). The opposite effect is observed in periods with higher HRV(mean NN), during which lower values of jitter are associated with an increased likelihood of exacerbation. Similarly, a positive association was observed between mean F0 and exacerbation during periods with moderate levels of HRV(mean NN).

**Fig. 2:**
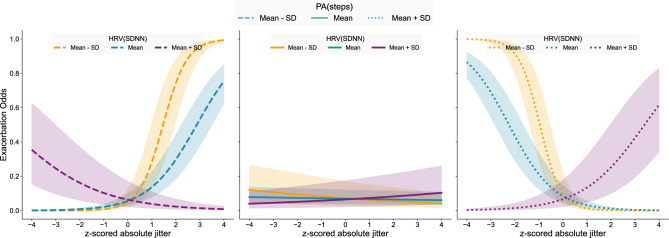
The interaction between HRV(SDNN), PA(steps), and z-score normalized absolute jitter in speech with respect to exacerbations. The shaded regions surrounding each curve denote the 95% confidence intervals.

Although no significant three-way interactions were detected for symptom scores, the fact that the same phonation features had significant two-way interactions with both HRV and physical activity indicates the importance of multimodal data. The interaction plots in Fig. 2 depict the interplay between absolute jitter, HRV(SDNN), and PA(steps) with respect to the likelihood of exacerbations.

## Discussion

When air is pushed through the vocal folds during speech production, it lends useful information about the functional limitations of people’s respiratory airways. Although many researchers are turning towards deep learning to predict COPD outcomes from speech^20,25,28^, these techniques do not offer interpretable and explainable results that could inform future digital biomarkers. Others have examined manually extracted speech features as we do in our work^19,24^, often with a focus on overall predictive accuracy; however, there are a few studies that report on associations between speech features and outcomes. For example, Mohamed and el Maghraby^17^ observed that jitter and shimmer were inversely correlated with spirometry metrics like FEV1 and FVC, while Farrús et al.^18^ observed significantly slower speaking rates among COPD patients compared to healthy controls after physical activity.

Our study extends this literature by examining the associations between altered vocal characteristics and COPD outcomes, namely daily symptom score and exacerbations, during everyday living. We observed that categories of speech features reflecting different aspects of speech production exhibited unique relationships with COPD outcomes. Phonation features such as jitter, shimmer, and pitch and amplitude perturbations were found to have lower values when patients exhibited higher symptom scores or likelihood of an exacerbation. This trend may be attributed to increased vocal fold tension and rigidity due to heightened respiratory effort and stress^29^, resulting in a more stable but strained and higher-pitched voice. Our findings also reveal that prosodic features, specifically mean fundamental frequency (mean F0) and its standard deviation (std F0), were positively associated with exacerbations. On the other hand, formant frequencies like mean F2 showed a negative association with symptom scores. These observations diverge from previous research on acoustic measures for identifying pathological speech^22,30^. A key distinction in our study is the use of natural and unprompted speech, reflective of conditions in a remote patient monitoring setup. In free-living scenarios, patients may avoid speaking under extreme distress, so the observed vocal characteristics during this period likely stem from compensatory mechanisms where patients unconsciously adjust their vocal production to maintain intelligibility. Additionally, the negative association of mean F2 with symptom scores may result from reduced articulatory precision due to discomfort, leading to less distinct vowel articulation and lower F2 frequencies ^31^.

We also extend the literature by showing that the relationships between speech features and COPD outcomes are influenced by physiological covariates inherent to everyday living, such as HRV and physical activity. Our interaction models revealed that during periods of higher HRV, there was a stronger association between lower jitter and worsened COPD outcomes; the same held true for lower shimmer. This suggests that patients with more adaptable cardiac autonomic function, as indicated by higher HRV, may experience a more stable vocal fold pattern, even under respiratory distress. However, when HRV is lower—a state linked to increased susceptibility to adverse COPD outcomes^11^—the relationship was reversed. In these instances, increased jitter correlated with higher symptom scores and a greater likelihood of exacerbations, consistent with findings from prior studies. This variation emphasizes the complex interplay between respiratory effort, autonomic regulation, and vocal production in COPD patients. The impact of physical activity on these interactions further highlights the vulnerability of patients with low activity levels. During periods of reduced activity, the association between both jitter and HRV with exacerbation risk became more pronounced, consistent with previous research identifying low physical activity as a marker of increased exacerbation risk^32,33^.

By analyzing the combined influence of speech characteristics, HRV, and physical activity on COPD outcomes, our study is the first to provide comprehensive insights into the physiological manifestation of COPD. Although the effect sizes of the interaction terms were typically modest, as expected in free-living conditions with real-world noise, the consistency and statistical significance of the associations highlight that physiological context matters when interpreting vocal markers. This multimodal approach not only provides a holistic view of COPD but also synthesizes and reconciles findings from previous studies that have individually linked various physiological parameters with COPD severity, thereby advancing the field with a unified perspective on disease pathophysiology. It paves the way for developing composite digital biomarkers that combine multiple data sources to predict adverse outcomes such as exacerbations. These digital biomarkers could provide real-time, actionable insights into a patient’s health status, facilitating proactive management and personalized interventions. Additionally, our findings suggest that incorporating HRV metrics and step count into speech analysis models could potentially enhance the predictive power for symptom exacerbation and severity, enabling a more precise assessment of a patient’s condition.

Our findings also underscore the potential of off-the-shelf wearables for continuous respiratory health monitoring. These devices could provide accessible, cost-effective alternatives to traditional lung function tests, which are often burdensome and limited in availability. The adoption of wearable technology in remote patient monitoring could enable patients to actively track their symptoms and severity while allowing clinicians to remotely observe and manage patient health. This continuous monitoring approach could enhance early detection of exacerbations, prompt timely medical interventions, and ultimately improve patient outcomes. Moreover, it has the potential to reduce healthcare costs by minimizing the need for frequent in-person visits and hospitalizations, making it a promising avenue for future research and development in COPD management.

We acknowledge several limitations in our study. Exacerbations are clinically defined according to self-reported symptoms, but the subjective nature of those self-reports may have implications on the robustness of our results. Although comparisons with objective pulmonary function tests (PFTs) would strengthen the validity of our results, prior literature has noted that lung function tests can be burdensome and inaccurate, particularly when done at home without supervision^34–36^. Using self-reported symptoms yielded a more comprehensive and longitudinal assessment of the COPD condition, but future work could investigate accessible objective measures like at-home spirometry with automated coaching.

Another limitation of our study is the presence of noise in both speech and physiological data. We incorporated several measures to ensure quality in each stream of data. For speech, the SileroVAD voice activity detector^37^ was used to exclude audio segments lacking discernible speech or containing excessive noise. For HRV, we relied on Samsung’s proprietary heart rate tracking algorithm combined with filtering techniques to eliminate implausible inter-beat intervals and account for missing values. Similarly, Samsung’s step-counting algorithm likely includes safeguards against misclassifying non-periodic movements as steps. Despite these measures, residual noise may have affected the accuracy of our measurements, so future studies could benefit from more advanced preprocessing techniques or alternative metrics that are robust to noise.

A further consideration is the potential influence of language on the observed relationships between speech and COPD outcomes. Although the acoustic features analyzed in this study primarily capture low-level aspects of speech production, cross-linguistic variation in phonemic structure, prosodic patterns, and speaking conventions may influence their distributions. Consequently, the strength or direction of the associations we uncovered may differ across languages. On a related note, the relatively small sample size for exacerbations may limit the generalizability of our findings. Our models accounted for demographic confounders to mitigate the influence of potential biases in the cohort, but we acknowledge that a larger and more diverse sample would improve the statistical significance and generalizability of our conclusions.

## Methods

### Subjects

A total of 35 patients (18 females, mean age: 69.6 ± 9.2) diagnosed with COPD were recruited from three hospitals in Toronto, Canada to participate in our study. Participants were informed that they could withdraw from the study at any point in time. Those who required hospitalization or failed to adhere to the study protocol were de-enrolled. Study participants remained in the protocol for an average of 198.9 ± 122.6 days. The analysis in this paper is restricted to those who were not de-enrolled and had more than 3 weeks of data, leaving us with 18 patients ranging in age from 55–93 years (6 females, mean age: 67.3 ± 8.5).

### Study design and data collection

The study protocol received approval from the Research Ethics Board at the University of Toronto under Protocol #41568. All participants provided informed consent, and the study was conducted in accordance with the Declaration of Helsinki. Participants collected data at home over the course of several months, with the total study period being approximately two years from the start date of the first participant to the end date of the last participant. Demographic data was collected at the start of the study, with participants reporting their sex, age, smoking history, and comorbidities in an electronic survey.

Participants were given a Samsung Galaxy Watch to collect data and a Samsung Galaxy Note smartphone with a pre-installed application to facilitate data transmission. Participants were asked to wear the watch during their daily activities and to charge it at night, during which the smartphone application would identify and upload new data files. The smartwatch recorded audio data from the microphone at a sampling rate of 44.1 kHz, heart rate from the photoplethysmography (PPG) sensor at a sampling rate of 100 Hz, and daily step count from the motion data. To preserve the smartwatch’s battery life, a 20% duty cycle was applied such that 2 minutes of data recording was followed by 8 minutes of idle time.

Participants also used the smartphone app to report the severity of their COPD symptoms every morning according to the London COPD Cohort Daily Symptom Questionnaire^26,27^, a clinically validated instrument that has been used in both clinical^27,38^ and technical^24,39^ studies to screen for COPD exacerbations. The questionnaire requires patients to reflect on the severity of the eight symptoms by selecting the ones that were “worse than usual on the previous day”. The questionnaire considers three major symptoms—change in sputum color, increased breathlessness, and increased sputum amount—with a score of 5. The remaining symptoms—cold, fever, increased wheezing, sore throat, and worsening cough—are considered to be minor symptoms with a score of 1. An exacerbation is defined as two consecutive days with a symptom score of 6 or above, indicating two consecutive days with at least one major and one minor symptom. COPD symptom score and exacerbations constitute the two outcome variables investigated in this paper.

**Table 6:** Overview of features used for analyzing sensor data.

Data	Category	Feature	Description
Speech	Phonation features^40^	HNR	Harmonic sound to noise ratio
		jitter	Frequency perturbation across vocal fold cycles
		absolute jitter	Absolute frequency perturbation
		ddp jitter	Average absolute difference between the frequency differences of adjacent pitch periods
		ppq5 jitter	Five-point perturbation quotient for vocal fold frequency
		rap jitter	Relative average perturbation of vocal fold frequency
		shimmer	Variability of the peak-to-peak amplitude
		apq3 shimmer	Three-point amplitude perturbation quotient
		apq5 shimmer	Five-point amplitude perturbation quotient
		apq11 shimmer	Eleven-point amplitude perturbation quotient
		dda shimmer	Average absolute difference between the amplitudes of consecutive periods
	Prosodic features^41^	mean F0	Mean fundamental frequency
		std F0	Standard deviation of fundamental frequency
		mean F1	Average value of the first formant frequency (F1)
		mean F2	Average value of the second formant frequency (F2)
		mean F3	Average value of the third formant frequency (F3)
		mean F4	Average value of the fourth formant frequency (F4)
		mean formant	Average value of all the four formants
		delta_f^42^	Weighted average of the first four formant frequencies (F1, F2, F3, F4) based on their respective positions
		fdisp^43^	Formant dispersion, calculated as the average spacing between adjacent formant frequencies
		fitch_vtl^43^	Vocal tract length, calculated as the weighted average of the wavelengths of the first four formants
		mff^44^	Geometric mean of the first four formant frequencies
		pF^45^	Formant position, calculated as the average standardized deviation of the first four formant frequencies from their respective means
		vtl_delta_f^43^	Vocal tract length, calculated as the inverse of delta_f
	Syllabic nuclei^24^	pause rate	Total number of pauses divided by the duration of the segment
		phonation time	Total duration of syllables divided by the voiced segment duration
		speech rate	Total number of spoken syllables divided by the duration of the segment
Heart rate variability^46^		HRV(mean NN)	Average of all the normal-to-normal (NN) intervals
		HRV(SDNN)	Standard deviation of all the NN intervals
		HRV(pNN20)	Proportion of successive NN intervals that differ by greater than 20 ms
Physical activity		PA(step count)	Daily step count

### Data processing

As participants wore their smartwatches during their daily living, they may have produced non-speech sounds or encountered other ambient noises. To isolate useful speech segments from real-world audio data, we first removed intervals of silence by sliding a 50-ms window and discarding audio that fell below an overall intensity of −20 dB. The non-silent recordings were then split into 2-second windows with a 1-s step (50% overlap) and passed through a voice activity detector called SileroVAD^37^. SileroVAD is a neural network pre-trained on huge speech corpora including over 100 languages with various background noise and quality levels. The model takes a 20 ms audio frame to generate a speech probability score, which we then binarized using an empirically determined threshold of 0.7 to separate speech and non-speech segments. The binary labels for each frame were aggregated back into their original 2-second windows to identify longer segments of contiguous speech. We discarded all utterances shorter than 250 ms based on the observation that most meaningful speech utterances are longer than that duration^47^. If the combined duration of all utterances in a window was longer than 1.5 seconds (75% of the window), we considered that window as speech and retained it for further analysis. Across participants, these processing steps yielded an average of 122.6 $\pm$ 97.4 minutes of usable speech per day, from an initial average of 161.3 $\pm$ 123.2 minutes per day of audio.

After identifying speech segments, we extracted three categories of speech features that are known to be correlated with health^48,49^: (1) phonation features, which capture the temporal and amplitude variations in the vibration of the vocal cords; (2) prosodic features, which measure the pitch and loudness of each frame; and (3) syllable nuclei features, which relate to the speaking rate according to syllables. Table 6 lists and defines all of the features we used to characterize the change in vocal characteristics of a patient. Because COPD symptom severity was self-reported daily in our dataset, we computed the daily average of each feature to generate a single feature vector summarizing a whole day of speech audio. This step also reduced short-term variability caused by transient factors such as background noise, yielding robust and stable features that better reflect the gradual changes patients typically exhibit over daily timescales^26^.

We also examined the interplay between physical activity and heart rate variability (HRV) as physiological covariates with speech to characterize vocal changes and COPD severity more comprehensively. For physical activity, we used the aggregate daily step count calculated by Samsung’s proprietary algorithm; we call this covariate PA(steps). Daily step count data were available for an average of 136.7 $\pm$ 86.4 days per participant. For HRV, we first processed the 1-Hz heart rate data reported by Samsung’s proprietary algorithm. PPG data collected in the wild contains noise from several sources, including motion artifacts, wristband looseness, and environmental light^50^. As a result, the heart rate information provided by the smartwatch may have missing values that can impact derived features. We only considered heart rate data segments with a minimum length of 45 s in order to preserve the low-frequency component of the HRV signal (0.04–0.15 Hz)^51^. To calculate HRV, the heart rate data was converted to RR intervals according to the hyperbolic relationship between the two (HR $\times$ RR interval = 60,000)^52^. We then used a range-based filter to remove physiologically impossible values below 350 ms and above 1200 ms. Finally, we calculated three HRV metrics informed by prior literature ^53,54^ in each segment: HRV(mean NN), the mean of normal RR intervals; HRV(SDNN), the standard deviation of normal RR intervals; and HRV(pNN20), the percentage of consecutive normal RR intervals that differed by more than 20 ms. Similar to speech and activity data, these metrics were averaged across a day to represent daily HRV. Participants had an average of 85.5 $\pm$ 67.0 days of usable HRV data, compared to 138.1 $\pm$ 87.7 days prior to data processing.

### Statistics

Given the large number of candidate speech features and interaction terms, we adopted a two-step procedure to identify a subset appropriate for inclusion in the multivariate models alongside HRV and physical activity data. First, we fit univariate linear mixed-effects models for all speech features, with either COPD symptom score or exacerbation as the outcome variable. Next, we calculated variance inflation factor (VIF) values to determine whether the inclusion of the significant features and their corresponding interaction terms would create multicollinearity in the ultimate multivariate model. Starting with the full model including all of the terms, we iteratively removed those with the highest VIF values and re-estimated the model until all retained terms had VIFs were below 20.

Once we had our final set of statistically significant and uncorrelated speech features, we generated a multivariate model that included those features along with our four physiological covariates—three HRV metrics and daily step count—as modifiers of vocal changes associated with COPD outcomes. The multivariate model also included two-way interactions between the significant speech features and each of the physiological covariates, along with three-way interactions involving all three data types. For physiological covariates that appeared in multiple significant interaction terms, we conducted a moderation analysis inspired by Bauer and Curran ^55^ to examine whether the direction or strength of association between speech features and outcomes was affected by different covariate levels. We first stratified the data according to whether the notable physiological covariate fell within one of four intervals: < Mean - SD, [Mean - SD, Mean), [Mean, Mean + SD), and > Mean + SD. We then fitted separate univariate models for the corresponding speech features in the significant interaction terms.

All analyses were adjusted for three demographic confounders selected from prior literature^56^: age, sex, and smoking history. We report the $\beta$-coefficients for symptom score relationships and odds ratios for exacerbation relationships. We also include the 95% confidence intervals (CI) and p-values for these relationships with statistical significance being set at $p<.05$. All analyses were conducted in R-3.5.1 using the lmer^57^, tidyverse^58^, and interactions^59^ packages.

## Data Availability

On reasonable request, the dataset generated during and/or analyzed in this paper is available from the corresponding author.
